# A Novel Complex Networks Clustering Algorithm Based on the Core Influence of Nodes

**DOI:** 10.1155/2014/801854

**Published:** 2014-03-10

**Authors:** Chao Tong, Jianwei Niu, Bin Dai, Zhongyu Xie

**Affiliations:** ^1^School of Computer Science and Engineering, Beihang University, Beijing 100191, China; ^2^School of Computer Science, McGill University, Montreal, QC, Canada H3A 0E9

## Abstract

In complex networks, cluster structure, identified by the heterogeneity of nodes, has become a common and important topological property. Network clustering methods are thus significant for the study of complex networks. Currently, many typical clustering algorithms have some weakness like inaccuracy and slow convergence. In this paper, we propose a clustering algorithm by calculating the core influence of nodes. The clustering process is a simulation of the process of cluster formation in sociology. The algorithm detects the nodes with core influence through their betweenness centrality, and builds the cluster's core structure by discriminant functions. Next, the algorithm gets the final cluster structure after clustering the rest of the nodes in the network by optimizing method. Experiments on different datasets show that the clustering accuracy of this algorithm is superior to the classical clustering algorithm (Fast-Newman algorithm). It clusters faster and plays a positive role in revealing the real cluster structure of complex networks precisely.

## 1. Introduction

With the population of information networks and the discovery of the small world effect and the scale-free characteristic, research on complex networks has become a trend. Complex network study involves graph theory, statistical physics, computers, ecology, sociology, and economics [1]. Complex networks cover a variety of biology networks, the Internet/WWW networks, technology networks, social networks (such as the disease spreading networks and human relationships), and so on.

One of the most important features in complex networks is the cluster structure. Many studies have shown that some networks have cluster structures other than a large number of nodes only randomly linked. Heterogeneity has been found in many real-world networks. The heterogeneity of complex networks is embodied in more connections in similar types of nodes, while different types of nodes have fewer connections. These subgraphs with similar types of nodes and their connections are called “clusters.”

Clustering algorithm plays a basic role in studying the cluster structure of complex networks. It has not only important theoretical significance in researching complex network topology, understanding the network function, revealing hidden laws, and predicting the network behavior but also broad application prospects. Clustering algorithm has been applied to the social network analysis, biological network analysis, search engine, spatial data clustering and image segmentation, and many other areas [2].

According to the analysis strategy, complex network clustering methods are divided into optimization methods and heuristic methods. The earlier clustering algorithms like spectral method [3–6] and the Kernighan-Lin algorithm (KL algorithm) [7] are optimization methods. Spectral method, derived from the early resolution of the graph partition problem, uses the quadratic optimization techniques to minimize a predefined “cut” function. A partition with minimum “cut” is considered to be an optimal network partition. With rigorous mathematical theories, the spectral method is widely used in graph partitioning and spatial points clustering. However, high reliance on the prior knowledge and adoption of bipartite recursion strategy makes it inadequate in complex multiple clusters networks.

KL algorithm is also based on the idea of graph partition, which aims at minimizing the difference between the number of intercluster connections and internal connections. By continuously adjusting clusters, the algorithm chooses and accepts the candidate solutions that can get the minimization of the objective function. KL algorithm, very sensitive to the initial solution and highly dependent on the prior knowledge, often gets local optimal results.

Girvan and Newman proposed GN algorithm [8], which uses heuristic strategy by repeatedly identifying and removing the connections between clusters. GN is a big time-consuming and space-consuming algorithm, resulting from the complexity of the edge betweenness calculation (*O*(*m* × *n*)). Thus, it is difficult to perform well in a large network.

Based on the Maximum Flow-Minimum Cut Theorem, Flake et al. proposed a heuristic clustering algorithm, the Maximum Flow Community [9] (MFC algorithm). By calculating the minimum cut sets, MFC algorithm identifies intercluster connections that give rise to the network “bottleneck” and gradually split the network into cluster units by removing the connections between clusters. However, the MFC algorithm performs clustering based on connections and cannot be applied to the network with heterogeneous nodes.

Newman proposed a fast clustering algorithm based on local search [10], the Fast-Newman. The algorithm is an optimizing algorithm aiming at maximizing the network modular evaluation function (*Q* function) that Newman put forward in the same year. The *Q* function, denoted by the difference between the number of connections within a cluster and the expected number of connections in a random state, is used to show the pros and cons of the cluster structure. Larger *Q* value means a better clustering structure.

Based on the FN algorithm, Guimera and Amaral similarly adopted the *Q* function as the optimization objective function and proposed a complex networks clustering algorithm based on simulated annealing (SA), the GA algorithm [11]. The algorithm evaluates candidate solutions by calculating the corresponding *Q* function value and calculates the probability of accepting a candidate solution through the SA model. GA algorithm has the ability to find the global optimal solution and thus has good clustering performance.

Although optimization algorithms based on the *Q* function perform well in the community clustering, a number of issues remain unresolved due to the unpredictability of complex networks and the biased characteristic of *Q* function. Consider the following.

1. Since the community detection results through the clustering algorithms based on optimization depend on the objective function to be optimized, “biased” objective function will inevitably lead to “biased” solution. The *Q* function currently widely used is a biased objective function [12], by which the results cannot completely and accurately reflect the real network structure. When the *Q* function reaches the global maximum, the clustering result is not optimal.
2. With larger scale of complex networks, the calculation of the objective function and the iterative process become more complex, resulting in more and more time and resources consumed.
3. Though the clustering algorithm based on heuristics method is able to handle the large-scale data in complex networks, compared to the optimization algorithm, it has lower clustering accuracy and cannot give high-precision clustering results.

To solve the above problems, we proposed a novel clustering algorithm based on the core influence of nodes. The algorithm combines heuristics method with optimization method. Its clustering process is designed to simulate the driven process of the cluster formation in sociology, to reflect the clustering process of nodes in the real network more accurately, and to achieve “no biased” precise clustering as far as possible.

The rest of this paper is organized as follows. In the next section, we introduce the clustering algorithm based on the core influence of nodes, then the experimental results and analysis are illustrated in Section 3, and, finally, a conclusion is drawn in Section 4.

## 2. Clustering Algorithm Based on the Core Influence of Nodes

The basic idea of the clustering algorithm based on the core influence of nodes is to identify the nodes with core influence based on the betweenness centrality theory, build the core structure of clusters with these nodes in the complex network through the evaluation function, and, finally, cluster the remaining nodes in the network using optimizing methods. Thus, clusters of the whole network can be obtained.

### 2.1. The Definition of the Core Influence of Nodes

The core influence of nodes in complex networks is denoted by the centrality of nodes. Centrality refers to the use of metric methods to evaluate the center position of a node in the network. It describes whether there are cores, how many cores there are, and how these cores are in the network.

Centrality has many definitions in complex networks, such as the degree centrality, the compactness centrality, the betweenness centrality, and the flow betweenness centrality. In order to reveal the role the nodes play in the transferring process of information, material, and energy in the complex network, this paper uses the betweenness [13] (the number of geodesics through the node) to define the centrality and the core influence of nodes.

Geodesic is defined as the path with least edges between two nodes. Thus, betweenness centrality of node *x* [14] is defined as

(1) $$\begin{array}{c} C_{B}\left(x\right)=\frac{2\sum_{i<j}^{}g_{ij}\left(x\right)}{\left(n-1\right)\left(n-2\right)g_{ij}}, \end{array}$$

where *g*_*ij*_ is the total number of geodesics between node *i* and *j*,  *g*_*ij*_(*x*) is the number of geodesics through the node *x* between node *i* and *j* (the betweenness of node *x*), and (*n* − 1)(*n* − 2)/2 is the maximum value of the betweenness of the node *x* (any geodesic between other two nodes goes through the node *x*).

Betweenness centrality partially describes the core influence of nodes in complex networks. However, betweenness centrality itself is a global evaluation parameter, which cannot accurately describe the relative influence of nodes in the local environment, especially in large-scale complex networks. Therefore, combining the betweenness centrality and local clustering features of nodes, the core influence of nodes is denoted as

(2) $$\begin{array}{c} C_{A}\left(x\right)=\frac{C_{B}\left(x\right)}{2E_{x}/\left(k_{x}\left(k_{x}-1\right)\right)}, \end{array}$$

where *C*_*B*_(*x*) is the betweenness centrality of the node *x*, *k*_*x*_ represents the number of neighbor nodes of the node *x*, *E*_*x*_ denotes the total edges between the neighbors.

The definition of the core influence above accurately describes how important a node is in its clustering environment. Higher core influence of a node indicates higher contribution and heavier load in the information dissemination process in a complex network. Meanwhile, different from the simple degree centrality, a node with the highest core influence is not probably the node with the maximum degree or a topological center in the network structure.

### 2.2. The Determination of Cluster's Core Structure

In complex networks, the core structure of clusters is usually not only a simple single node with high core influence but possibly also a certain structure composed of several active nodes with high influence [15]. In order to determine the core structure of a cluster by the core influence of nodes, the *K* function is used here as the evaluation function to determine the nodes that compose the core structure of clusters.

The goal of the *K* function is to determine whether the node can become the core of an independent cluster. It compares the actual connections and expected connections between a node with high core influence and the cluster it belongs to. The function is defined as

(3) $$\begin{array}{c} K\left(i\right)=\frac{m_{i}}{\left(d_{i}/d\right)\times \left(\left(d_{q}-d_{i}\right)/\left(d-d_{i}\right)\right)\times m}, \end{array}$$

where *m*_*i*_ is the number of edges between node *i* and other nodes in the cluster that node *i* belongs to, *m* denotes the total number of edges in the whole network, *d*_*i*_ represents the degree of node *i*, *d*_*q*_ is the sum of degrees of nodes in the cluster, and d denotes the sum of degrees of nodes in the whole network. According to the definition of *K*(*i*), there is a higher probability that a node becomes the core of an independent cluster when its *K*(*i*) is smaller; while a node attached to a rather larger *K*(*i*) plays a more influential role in its current cluster.

According to Fortunato and Barthélemy's study of the *Q* function's value range on a large number of real datasets [12], it can be estimated that the value range of *K* function is [1.96, 2.71] when a node is thought to be the core of an independent cluster. Besides, according to the Pareto rule, 20% of the nodes in the network with the highest core influence determine the main cluster structure framework. Plenty of datasets reveal that 20% of the nodes in the network with the highest core influence can determine the core cluster structure framework after being evaluated by *K* function, which is consistent with the Pareto rule.

### 2.3. Clustering Algorithm

After determining the core cluster structure, the algorithm clusters the remaining nodes by optimizing method. The remaining nodes are centralized by rearranging all nodes regarding their core influence. A “centralized” network can thus be obtained, where nodes are arranged from inside to outside. The objective “centralization function” that reflects the level of centralization is then defined as

(4) $$\begin{array}{c} C_{A}^{g}=\frac{\sum_{x\in W}^{}\left(C_{A}^{*}-C_{A}\left(x\right)\right)}{\left(n-1\right)\max\left(C_{A}^{*}-C_{A}\left(x\right)\right)}, \end{array}$$

where *W* represents a complex network and *C*_A_* = max⁡_*x*∈*W*_ *C*_*A*_(*x*) represents betweenness centrality of the node that has the maximum core influence.

The objective function shows that if all nodes have the same core influence, which indicates that the network is noncore, then *C*_*A*_^*g*^ = 0; if the core influence of a node is 1, while other nodes remain 0, *C*_*A*_^*g*^ = 1. Therefore, the higher the level of centralization of the network is, the greater the value of the objective function is.

The strategy to search and accept the candidate solution is as follows. Firstly, arrange all nodes descendingly according to their core influence. Then, change the structure of the cluster a node belongs to and then calculate the corresponding “centralization function.” And accept the candidate solution that maximizes the sum of the value of the whole network's “centralization function.” The process ends when all nodes are classified into their own respective cluster structure.

### 2.4. Algorithm Implementation

According to the algorithm, the actual steps of the clustering algorithm based on the core influence are as follows.

1. Sort all nodes by betweenness centrality in descending order {*n*_*i*_}, satisfying the requirement that when *i* < *j*, *C*_*i*_ > *C*_*j*_.
2. Set up three groups of nodes; *P*[*i*] represents that the node has been clustered, *Q*[*i*] represents that the node has not been clustered, and *R*[*i*] represents that the node is in cluster-controversy. Initially, all nodes are in *Q*.
3. Select the node *n*_1_ with the highest degree and all the nodes connected to *n*_1_ are defined as a cluster, while *n*_1_ is the core of Cluster 1 and all the nodes are classified into the node group *P*.
4. Judge whether *n*_2_ is the core. If *n*_2_ is not in the node group *P*, *n*_2_ is the core of Cluster 2.
5. If *n*_2_ is within the node group *P*, use the criteria function *K*. If *K* ∈ [1.96,2.71], *n*_2_ is the core of Cluster 2; otherwise, classify the adjacent nodes of *n*_2_ into the cluster where *n*_2_ is. Repeat step (4) and judge node *n*_3_, *n*_4_….
6. For all nodes connected to Cluster 2's core, classify those in *Q* into Custer 2 and transfer them into *P*, while transferring those originally of the *P* to *R*.
7. Traverse the nodes in *R* through the optimization function “centric level,” redetermine their respective cluster, and transfer the nodes from *R* to *P* after they have entered the selected cluster.
8. Return to step (4) and iterate and traverse all the nodes.

## 3. Experimental Results and Analysis

For more objective and comprehensive evaluation, the algorithm is tested on three datasets (Neural Network [16], Political Blogs [17], and Email [18]) of different sizes and properties. The results are analysed and evaluated using the Conductance and Expansion results evaluation function in network, the Community Profile [19] (NCP). The two functions are defined as

(5) $$\begin{array}{c} \text{Conductance}:f\left(S\right)=\frac{c_{S}}{2m_{S}+c_{S}},\\ \text{Expansion}:f\left(S\right)=\frac{c_{S}}{n_{S}}, \end{array}$$

where *c*_*S*_ represents the number of edges on the boundary of *S*, *m*_*S*_ denotes the total number of edges within the Cluster *S* and *n*_*S*_ is the total number of nodes in Cluster *S*. Lower value of the two evaluation functions signifies better clustering effect.

### 3.1. Neural Network Dataset Experiment

In this section, the algorithm is tested on the dataset “Neural Network.” The dataset is a complex network of neurons in a living system, where each node represents a complete and independent neuron and the edge denotes the connection between neurons. The properties of the network are shown in Table 1.

The number of nodes and edges and the diameter describe the overall size of the network. The average clustering coefficient, the number of triangles, and the average shortest path length describe the relative tightness of the network and how obvious the clustering feature is.

**Figure 1:**
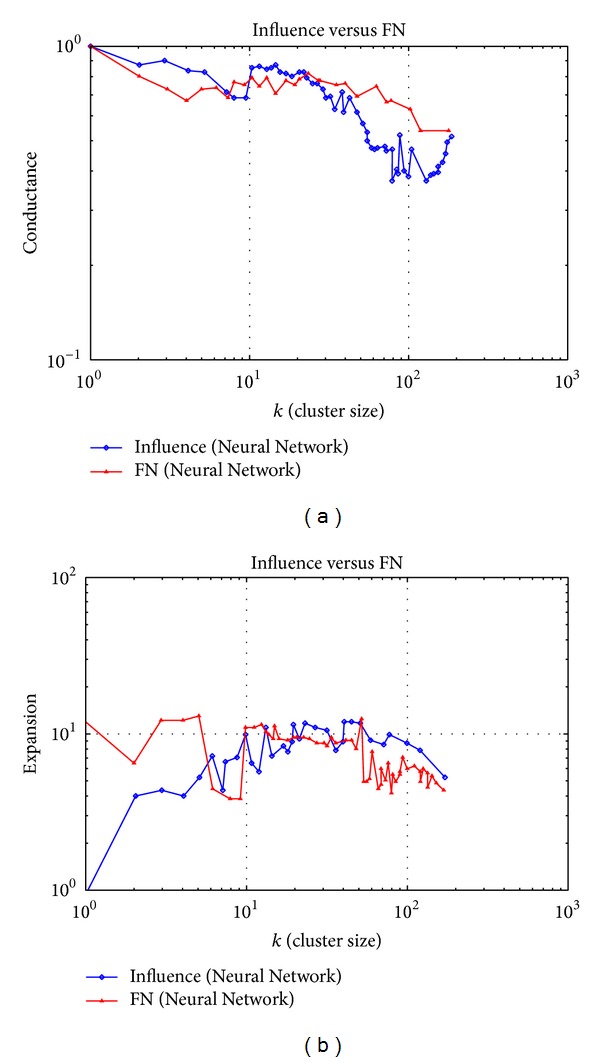
Evaluating values of the clustering effect of the Neural Network dataset.

The evaluating values of the clustering effect of two algorithms are shown in Figure 1. It can be calculated through the data shown in Figure 1(a) that the average Conductance of the influence algorithm is 0.540144, while the average of FN algorithm is 0.736532. The figure also shows that when the cluster size grows, the clustering effect of the influential algorithm improves, better than the FN algorithm. From Figure 1(b), it is calculated that the average value of the Expansion of the influence algorithm is 6.700091 and the average of FN algorithm is 8.205680. And, with the increase of the cluster size, the influence algorithm performs better than the FN algorithm in accuracy by 22.5%.

In this dataset, neurons have explicit functions and every neuron does not get global information. As a result, the FN algorithm cannot cluster precisely. The influence algorithm, proposed by us, however, digs out neurons with similar functional properties more precisely by considering the role every neuron plays in the process of information dissemination and gives the structural relationship among neurons of similar functions and among neurons clusters of different functions. The clustering results help medical researchers understand the mechanism of nervous system better so that they can analyze causes of neurological diseases and provide theoretical support for cures [20].

### 3.2. Political Blogs Dataset Experiment

In this section, the algorithm is tested on the dataset “Political Blogs.” The dataset is a political blog network in complex social networks, where each node represents a politician and the edge denotes the real social relations between them. Compared with the Neural Network dataset, Political Blogs dataset has a larger scale, where the number of nodes increases by 3.1 times and the number of edges increases by 7 times. So, the connections between nodes are closer, and the clustering coefficient and the number of short circuits (triangle closure) increase in the network. On the other hand, the average shortest path between nodes becomes longer, indicating that the increase of the tightness of relationships between nodes is limited, though the network is larger. The properties of the network are shown in Table 2.

**Figure 2:**
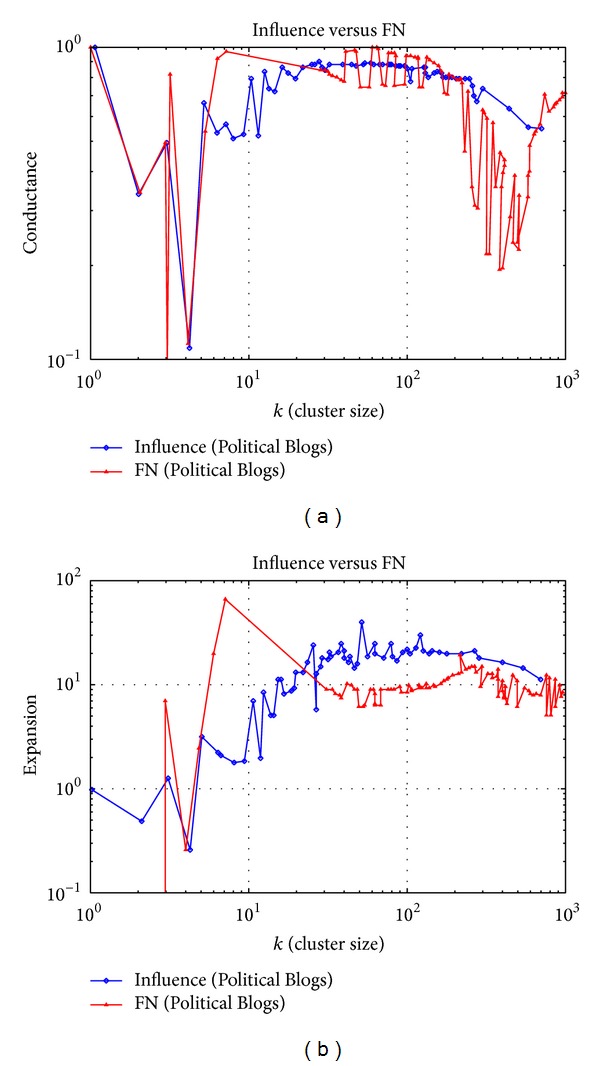
Evaluating values of the clustering effect of the Political Blogs dataset.

The evaluating values of the clustering effect of two algorithms are shown in Figure 2. It can be calculated through the data shown in Figure 2(a) that the average Conductance of the influence algorithm is 0.540144, while the average of FN algorithm is 0.736532. The value of Conductance of the influence algorithm is lower than the FN algorithm in 82.57% of all the cases. From Figure 2(b), it is calculated that the average value of the Expansion of the influence algorithm is 9.466124 and the average of FN algorithm is 16.379612. The clustering accuracy of the influence algorithm is better than the FN algorithm in 85.61% of all the cases.

In the comparison with Figure 1, the influence algorithm also keeps a high clustering accuracy, but the fluctuation range of the accuracy is wider. This indicates that as the complex network size increases, the local differences of the core influence and clustering feature of nodes increase. The influence algorithm reflects the local differences of nodes and reveals the significance of core influence nodes in the clustering process. It digs out the faction relationships among politicians more precisely.

The cluster analysis of the Political Blogs dataset by the influence algorithm is the theoretical basis of information diffusion and behavior spread in politics. For politicians, the clustering results help individuals to predict the support and resistance in the dissemination of their political opinion. The results also help predict the probability of the pass of a political proposal and even the election result.

### 3.3. Email Dataset Experiment

In this section, the algorithm is tested on the dataset “Email” in the social system, which is established by receiving and sending emails. Each node represents an email address and two nodes are connected when they have email exchanges in history.

Compared with the first two datasets, the “Email” dataset contains fewer nodes and sparser connections. Thus, it has lower clustering coefficient and larger average value of shortest paths. In this case, the locality of nodes is stronger and the probability for nodes to grasp global information is smaller. The properties of the network are shown in Table 3.

**Figure 3:**
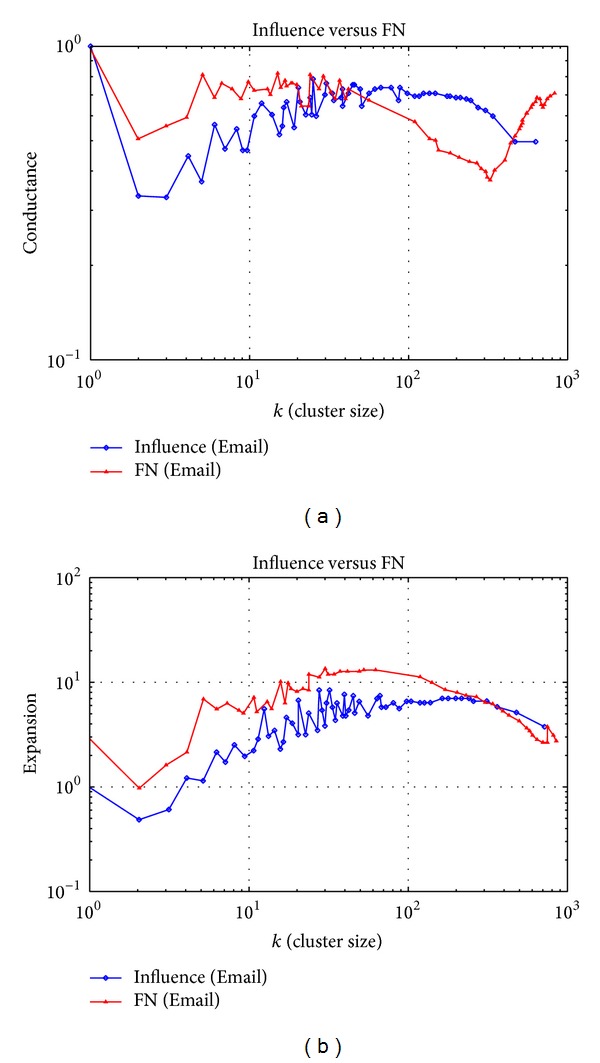
Evaluating values of the clustering effect of the Email dataset.

The evaluating values of the clustering effect of two algorithms are shown in Figure 3. It can be calculated through the data shown in Figure 3(a) that the average Conductance of the influence algorithm is 0.653043, while the average of FN algorithm is 0.664280. From Figure 3(b), it is calculated that the average value of the Expansion of the influence algorithm is 5.263551, while the average of FN algorithm is 4.619496. The clustering accuracy of the influence algorithm only has slight improvement compared to the FN algorithm. This is because the cluster coefficient becomes smaller with the expansion of clusters, leading to less and even the loss of difference of core influence among nodes. And less difference of core influence weakens the identity of the core cluster structure of the algorithm, indicating that the algorithm has limitations when processing sparse network with high homogeneity.

The experimental results on three datasets show that the clustering accuracy of the influence algorithm on large-scale complex networks increases variously compared to the FN algorithms. The effect is especially prominent for large-scale networks or networks with high heterogeneity. Studies have shown that when the size of a cluster is in the range of 50 to 100, the structure is relatively stable and real, and the effect of the clustering algorithm based on the core influence of nodes is much better than the FN algorithm in this interval.

## 4. Conclusion

In this paper, to solve the biasness in traditional clustering methods, we proposed an algorithm based on the core influence of nodes. On the basis of the core influence of nodes, the algorithm simulates the driven process of cluster formation in sociology. It absorbs the advantages of both heuristic and optimizing algorithms and reflects the real clustering process in a more accurate way. The clustering experiments on different datasets conclude that the clustering accuracy of this algorithm is superior to the classic clustering algorithm (FN algorithm) in complex networks. Meanwhile, this algorithm runs faster and plays a positive role in revealing the real cluster structure of complex networks.

Future studies can be conducted in two directions. Firstly, improve the algorithm based on the core influence of nodes to achieve higher accuracy and prove the “unbiased” nature of its clustering results. Secondly, optimize the iterative strategy of the algorithm to further improve the clustering efficiency when handling large-scale networks.

## Figures and Tables

**Table 1:** Neural Network dataset properties.

Properties	Values
Number of nodes	297
Average clustering coefficient	0.2924
Number of edges	2359
Diameter	5
Number of triangles	3241
Average shortest path length	2.4553

**Table 2:** Political Blogs dataset properties.

Properties	Values
Number of nodes	1222
Average clustering coefficient	0.3203
Number of edges	16717
Diameter	8
Number of triangles	101043
Average shortest path length	2.7375

**Table 3:** Email dataset properties.

Properties	Values
Number of nodes	1133
Average clustering coefficient	0.2202
Number of edges	5452
Diameter	8
Number of triangles	5453
Average shortest path length	3.6060
